# Potential use of mobile phones in improving animal health service delivery in underserved rural areas: experience from Kilosa and Gairo districts in Tanzania

**DOI:** 10.1186/s12917-016-0860-z

**Published:** 2016-10-07

**Authors:** Esron D. Karimuribo, Emmanuel K. Batamuzi, Lucas B. Massawe, Richard S. Silayo, Frederick O. K. Mgongo, Elikira Kimbita, Raphael M. Wambura

**Affiliations:** 1 Department of Veterinary Medicine and Public Health, Sokoine University of Agriculture, Morogoro, Tanzania; 2 Department of Surgery and Theriogenology, Sokoine University of Agriculture, Morogoro, Tanzania; 3 Department of Veterinary Microbiology and Parasitology, Sokoine University of Agriculture, Morogoro, Tanzania; 4 Department of Agricultural Education and Extension, Sokoine University of Agriculture, Morogoro, Tanzania

**Keywords:** Animal health delivery, E-based diagnostic system, Linking rural and urban areas, Tanzania

## Abstract

**Background:**

Sub-optimal performance of the animal health delivery system in rural areas is common in developing countries including Tanzania. However, penetration of mobile phones and availability of good road network and public transport systems offer opportunities for improving the access of rural communities to diagnostic and advisory services from facilities and expertise located in urban areas. A questionnaire survey on possession and use of mobile phones by pastoral and agro-pastoral communities in Kilosa and Gairo districts was carried out between November and December 2015. A total number of 138 livestock keepers from three villages of Chakwale (54), Mvumi (41) and Parakuyo (43) participated in the study. An e-based system was designed and tested to link rural communities with urban diagnostic facilities.

**Results:**

It was observed that the average number of phones possessed by individuals interviewed and household families was 1.1 ± 0.26 (1–2) and 3.5 ± 2.23 (1–10), respectively. It was further observed that out of 138 livestock keepers interviewed, 133 (96.4 %) had feature phones while 10 (7.2 %) of them possessed smartphones. Mobile phone is currently used to support livestock production by communicating on animal health in Parakuyo (18, 41.9 %), Mvumi (18, 43.9 %) and Chakwale (14, 25.9 %). Other contributions of mobile phones in livestock and crop agriculture observed in the study area include: exchange of livestock price information, crop price information, communicating on plant health/diseases, livestock extension and advisory services as well as crop farming extension and advisory services. We also designed and tested an e-based SUAVetDiag® system to support timely diagnosis of infectious disease conditions and prompt advice on case management in veterinary underserved areas.

**Conclusions:**

Availability of mobile phones in rural areas, in combination with supporting infrastructure and facilities in urban areas, has potential to stimulate local development and improving delivery of animal health and extension services. It is recommended that more development and refinement of the system should be conducted to ensure that this potential is tapped to revolutionalise delivery of animal health services in rural areas.

## Background

Since 2011, a Beef and Milk Value Chain project supported by the Norwegian bilateral grant to Tanzania through the Enhancing Pro-poor Innovations in Natural Resources and Agricultural Value Chains (EPINAV) programme was implemented in Kilosa and Gairo districts [1]. The overall objective of this project was to enhance beef and dairy animal productivity and marketing in pastoral settings in order to ensure constant supply of meat and milk and in doing so, promote livelihood security through greater participation of pastoral communities along the products value chains. The project targeted to introduce interventions focusing on improving productivity of pastoral and agro-pastoral cattle to optimal levels through improved animal feeding, particularly during the dry season, animal breeding and animal health.

The performance of animal health delivery systems in developing countries, including Tanzania, has been declining with time [2]. A number of factors contributed to this situation including decision of African governments to privatize animal health services, reluctance of private practitioners to operate in commercially unattractive under-served areas and lack of incentive packages and motivation for animal health providers in rural areas [3, 4]. Despite the high prevalence of diseases in rural areas, livestock keepers do not have supporting infrastructure to diagnose different disease conditions before treatment. The situation is aggravated by indiscriminate use of veterinary drugs including antibiotics that are readily available in livestock markets [5]. All these factors contribute to increased risk of presence of antimicrobial residues in animal products such as beef and milk as it has been reported in different studies [6, 7].

During the mapping out of key actors along the beef- and milk value chains in the EPINAV-funded project, it was realised that an opportunity of pastoral and agro-pastoral owning mobile phones could be exploited to improve delivery of animal health services as well as provision of extension services. This paper shares our experience which contributed to the design and testing of a mobile phone-supported SUA-VetDiag system that has potential to improve animal health and productivity, not only in the study area but also in Tanzania and beyond.

## Methods

### Study area

This work was carried out in Kilosa and Gairo districts of Morogoro region in Tanzania between November and December 2015. It involved conducting of questionnaire survey in two villages of Kilosa districts (Parakuyo and Mvumi) and one village of Gairo (Chakwale) district. Detailed information on location and geo-climatic features of the study districts has been described previously [6]. The study villages are inhabited by different tribes as summarized in Table 1.

**Table 1:** Study villages, inhabitants, main economic activities and mobile phones possession

District	Village (No. interviewed)	Type of participants and their main economic activities		Mobile phone possession						
		Livestock keeping inhabitants	Main economic activity	Level/No. phones	1 (%)	2 (%)	3–4 (%)	5–6 (%)	7+ (%)	Total
Kilosa	Parakuyo (43)	Maasai	Pastoralists	Individual	39 (90.7)	4 (9.3)	0 (0.0)	0 (0.0)	0 (0.0)	43 (100.0)
				Family	7 (16.3)	7 (16.3)	15 (34.9)	9 (20.9)	5 (11.6)	43 (100.0)
	Mvumi (41)	Sukuma	Agro-pastoralists	Individual	36 (90.0)	4 (10.0)	0 (0.0)	0 (0.0)	0 (0.0)	40 (100.0)
				Family	15 (46.9)	9 (28.1)	6 (18.8)	1 (3.1)	1 (3.1)	32 (100.0)
Gairo	Chakwale (54)	Kaguru and Wanguu	Agro-pastoralists	Individual	50 (96.2)	2 (3.8)	0 (0.0)	0 (0.0)	0 (0.0)	52 (100.0)
				Family	17 (45.9)	6 (16.2)	9 (24.3)	2 (5.4)	3 (8.1)	37 (100.0)
Overall	(138)	–	–	Individual	125 (92.6)	10 (7.4)	0 (0.0)	0 (0.0)	0 (0.0)	135 (100.0)
				Family	39 (34.8)	22 (19.6)	30 (26.8)	12 (10.7)	9 (8.0)	112 (100.0)

Selection of the study villages was purposively carried out to understand variation in access and use of mobile phones by different pastoral and agro-pastoral communities. Participants were members of the beef and milk value chain innovation platforms that were organized by the EPINAV funded project [8]. They were, therefore, inhabitants of the study villages owning cattle and small ruminants who voluntarily agreed to participate in research project activities. Previous studies in the study area showed that the majority of livestock keepers in Kilosa and Gairo districts do not have formal education (53.6 %); only 35.5 % had primary school education; 5.0 % had secondary school education and only 1.4 % had college education [9]. In addition to keeping cattle, the pastoral and agro-pastoral communities also keep goats and sheep.

### Design of the e-based support system

An explorative work was carried out to recognize opportunities available to support timely collection, submission, processing and provision of timely laboratory results on major infectious disease conditions affecting cattle in the study area. Using the opportunities identified, a short-term ICT programmer was contracted to design a digital system that could support early diagnosis and provision of laboratory results on disease conditions affecting cattle including advice on management of cases diagnosed. The system designed was also tested after supporting key requirements for the system to work, including training of livestock keepers on how to identify sick animals, collect, label, and submist specimens to the urban-based diagnostic facilities in Morogoro municipality.

### Data collection

Structured questionnaire survey was carried out to collect information on mobile phone possession at an individual and family level, type of mobile phones possessed and major uses of the phones in relation to the animal keeping and management activities as well as other socio-economic activities. Furthermore respondents were asked to report on mobile phone network coverage and perception on which network has better signals in their localities.

### Statistical analysis

Data collected were entered into an Excel database and, descriptive statistics computed using Epi Info statistical programme. Statistical significance between differences in means and proportions of various groups compared was carried out using the Mann-Whitney and Chi-squared tests, respectively at critical probability of 0.05.

## Results

### Mobile phone possession and use

**Fig. 1:**
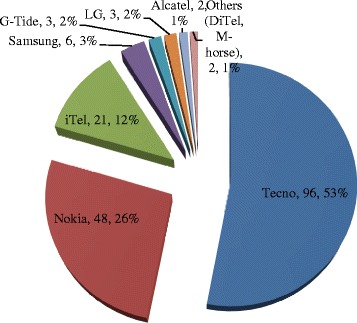
Different brands of mobile phones possessed by livestock keepers in Kilosa and Gairo districts, Tanzania

A total number of 138 livestock keepers from Parakuyo (43), Mvumi (41) and Chakwale (54) villages, as summarized in Table 1, participated in the interview. The number of mobile phones possessed by individuals interviewed as well as their family members is indicated in Table 1. At the individual level, the number of phones possessed by livestock keepers in the three study villages was comparable. The majority of respondents (>92 %) possessed one phone and only very few had two phones. At the family level, mobile phone possession ranged between one and ten phones per household. In terms of possession of multiple phones, there was statistically significant variation between villages and family members whereby livestock keepers at Parakuyo possessed significantly higher number of phones than those at Mikumi and Chakwale (*p* = 0.00023). The type of phones possessed by livestock keepers are shown in Fig. 1. It was further observed that that out of 138 livestock keepers interviewed, 133 (96.4 %) had feature phones while 10 (7.2 %) possessed smartphones. The study villages had good mobile phone coverage provided by either Tigo, Airtel or Vodacom mobile phone service providers.

**Fig. 2:**
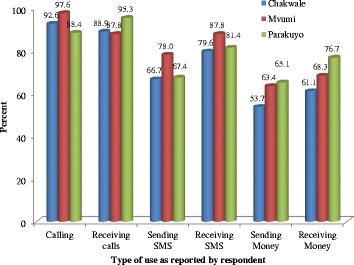
Different uses of mobile phones as reported by livestock keepers in the study villages

Mobile phones possessed by livestock keepers were reported to be used for different purposes as indicated in Fig. 2. Overall, the majority of respondents reported to use their phones for making and receiving calls followed by receiving Short Messages Services (SMS). Other uses reported by respondents were sending SMS, receiving money and also sending money. There was clear variation between study villages in the uses reported by the pastoralists at Parakuyo village leading in receiving calls as well as; receiving and sending money while the agro-pastoralists at Mvumi and Chakwale villages were good at making calls.

### How mobile phones are used to support livestock keeping and crop farming

Specific uses of mobile phones to support livestock keeping and crop farming are summarized in Table 2. There was variation based on location with pastoral livestock keepers at Parakuyo village using mobile phones more in communicating matters related to livestock keeping and selling than crop farming. There was also variation between agro-pastoral communities with those at Chakwale village reporting more use of mobile phones in crop agriculture compared with those at Mvumi village. It was further observed that more than 97 % of the respondents who participated in this study preferred to use mobile phones in receiving extension and advisory services related to livestock keeping.

**Table 2:** The current use of mobile phones in supporting livestock and crop agriculture in study villages

Use	Village	No. respondents	Percent
Exchange livestock price information	Parakuyo (*n* = 43)	17	39.5
	Mvumi (*n* = 41)	17	41.5
	Chakwale (*n* = 54)	16	29.6
Exchange crop price information	Parakuyo (*n* = 43)	1	2.3
	Mvumi (*n* = 41)	3	7.3
	Chakwale (*n* = 54)	13	24.1
Communicating on animal health/diseases	Parakuyo (*n* = 43)	18	41.9
	Mvumi (*n* = 41)	18	43.9
	Chakwale (*n* = 54)	14	25.9
Communicating on plant health/diseases	Parakuyo (*n* = 43)	1	2.3
	Mvumi (*n* = 41)	5	12.2
	Chakwale (*n* = 54)	11	20.4
Livestock extension and advisory services	Parakuyo (*n* = 43)	10	23.3
	Mvumi (*n* = 41)	18	43.9
	Chakwale (*n* = 54)	17	31.5
Crop farming extension and advisory services	Parakuyo (*n* = 43)	6	14.0
	Mvumi (*n* = 41)	8	19.5
	Chakwale (*n* = 54)	17	31.5
Like to receive information on livestock extension and advisory services using mobile phones	Parakuyo (*n* = 43)	40	93.0
	Mvumi (*n* = 41)	41	100.0
	Chakwale (*n* = 54)	53	98.1

### Opportunities for supporting an e-based system to support diagnostic and advisory services on livestock health

The initial mapping of actors along the beef and milk value chains identified the following opportunities and infrastructure that had potential to support prompt diagnosis and advice on case management of livestock diseases in the study area:

- Availability of public and private animal health diagnostic facilities in urban areas of Morogoro. The study specifically identified Sokoine University of Agriculture (SUA) Animal Hospital located at the Faculty of Veterinary Medicine (public) and Makanyaga Veterinary Clinic (private) as potential support of the system;
- Reliable road network connection between rural and urban areas. The Parakuyo village has a rough road connecting it to Morogoro municipality via a tarmac road from Melela along the Morogoro-Iringa highway. On the other hand, Mvumi has a tarmac road connecting it to Morogoro via Dumila along the Dodoma-Morogoro highway;
- There is reliable public transport between Parakuyo/Mvumi villages and Morogoro municipality;
- Each study village has one livestock extension officer who could support case management once proper diagnosis is made;
- The livestock farmers were able to collect appropriate specimens and samples including labeling and packaging them after training by the project staff.

### Designed e-SUAVetDiag®System and how it operates

**Fig. 3:**
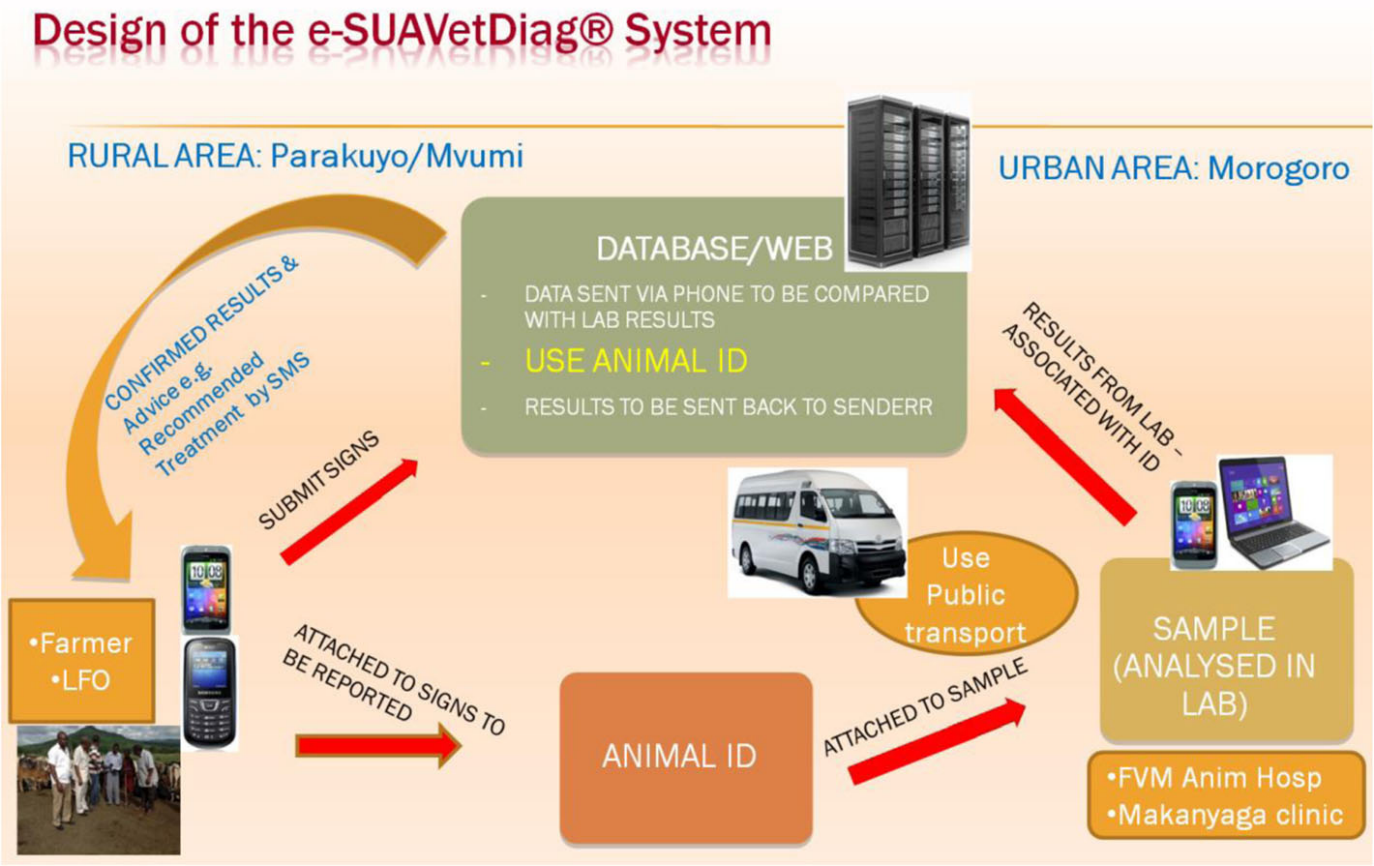
The e-based diagnostic system designed to support prompt diagnosis and advice to livestock keepers

Given availability of the above opportunities, the project designed in collaboration with SUA-based ICT programmer an e-based system (Fig. 3). Livestock keepers in collaboration with Livestock Field Officer (LFO) collect and label samples from sick animals, transport (using public transport system) the samples to SUA Animal hospital and Makanyaga Veterinary clinic for diagnosis. The farmers were trained to collect samples in duplicates so that one sample is processed by SUA Animal hospital while the second sample is processed by Makanyaga Veterinary clinic. While the samples are on transit, a sender conveys an SMS via mobile phone to a database that automatically informs a laboratory technician on samples sent, the bus and time the samples are expected to arrive at the bus stop in Morogoro Municipality. The samples are then collected by a receiving person from SUA Animal hospital who distributes the aliquots to both SUA Animal hospital and Makanyaga Veterinary clinic. Laboratory results, advices, recommendations and treatments are coded in a database. Coded data is then sent back to sender and LFO in form of SMS via their mobile phones (Fig. 3).

Testing of the system proved that the system could support diagnosis of specimens submitted within six to 12 h. It was further observed that livestock keepers at Mvumi village were more willing to use the system than their counterparts at Parakuyo village.

## Discussion

Findings of the current study indicate that mobile phones have considerably penetrated rural areas of Tanzania. For the pastoral and agro-pastoral communities, the mobile phones are already being used as valuable tools for communication and exchange of information related to animal health, livestock keeping and crop farming as well as other livelihood matters. These findings tally with other studies conducted within Tanzania [10–12] as well as elsewhere [13, 14]. This work has also indicated that although the majority of livestock keepers own one phone, some also have two phones and there are, on average, three phones in each household. Such findings indicate the existence of a potential use of the mobile phones to support the livestock keepers particularly those living in rural areas that are seriously underserved in terms of extension and advisory services for livestock and crop agriculture. This is also supported by findings of the present study where more than 97 % of the respondents interviewed were willing to receive extension support via their mobile phones.

This study also found that the majority of rural-based communities in the study area prefer to use mobile phones for making and receiving calls as well as receiving and sending SMS (Short Message services). This has implication on type of applications that should be designed by ICT programmers if these tools were to be considered to support rural-based communities. The programmers should consider using appropriate technologies and tools such as the Interactive Voice Response (IVR) and SMS-based technologies to develop tools and applications that can be easily adopted by the community-based livestock keepers. These technologies are believed to be economical and support prompt interaction and communication for matters related to their needs [15].

The observations of some mobile phone brands dominating the market such as the Tecno^®^ has also been seen and reported in other parts of Tanzania [16]. However there was variation in the work by Msuya [16] where Samsung^®^ ranked second compared to Nokia^®^ which was third. This difference may be attributed to the type of communities covered as the current study dealt with rural-based communities in Kilosa and Gairo districts while Msuya [16] focused on employees in the education sector in Kilimanjaro region. Informal discussion with livestock keepers interviewed during the current study found that the livestock keepers have some key mobile phone features they prefer such as a hardy phone that can withstand challenging environment such as dusty and rainy conditions as well as ability to sustain power for longer period in absence of regular battery charging facilities.

## Conclusion

The present studies in which e-SUAVetDiag system developed for mobile-phone based infectious disease diagnosis and animal health advisory service in the study area have highlighted the potential of mobile phone use for improvement of animal health service delivery. To the best of our knowledge, this is the first system that has been designed to support animal health diagnostic and telecare in Tanzania and East and Central Africa where experts based in urban centres can provide virtual advisory support to rural-based areas that are seriously underserved in terms of extension and animal health delivery services [9]. It is believed that if improved, the system has potential to revolutionalize delivery of animal health services in many parts of rural Tanzania as well as in other countries.

## Abbreviations

ANOVA: Analysis of variance
EPINAV: Enhancing Pro-poor Innovations in Natural Resources and Agricultural Value Chains
ICT: Information and Communications Technology
LFO: Livestock Field Officer
SMS: Short Messages Service
SUA: Sokoine University of Agriculture
